# Complete resolution of steroid-resistant organizing pneumonia associated with myelodysplastic syndrome following allogeneic hematopoietic cell transplantation

**DOI:** 10.1186/2193-1801-3-3

**Published:** 2014-01-02

**Authors:** Takeru Asano, Nobuharu Fujii, Daigo Niiya, Hisakazu Nishimori, Keiko Fujii, Ken-ichi Matsuoka, Koichi Ichimura, Toshihisa Hamada, Eisei Kondo, Yoshinobu Maeda, Yasushi Tanimoto, Katsuji Shinagawa, Mitsune Tanimoto

**Affiliations:** Department of Hematology, Oncology, and Respiratory Medicine, Okayama University Hospital, Okayama, Japan; Division of Transfusion, Okayama University Hospital, Okayama, Japan; Department of Pathology, Okayama University Hospital, Okayama, Japan; Department of Dermatology, Okayama University Hospital, Okayama, Japan

**Keywords:** Organizing pneumonia, Myelodysplastic syndrome, Sweet’s syndrome, Allogeneic hematopoietic cell transplantation

## Abstract

Pulmonary complications in patients with hematological malignancies are often caused by infection but are sometimes associated with an underlying disease such as organizing pneumonia (OP). Here, we report a case of life-threatening steroid-resistant OP associated with myelodysplastic syndrome (MDS) and successfully performed allogeneic hematopoietic cell transplantation (HSCT). A 33-year-old female with refractory anemia with excess blasts-1 that had progressed from refractory anemia with ringed sideroblasts and concomitant Sweet’s syndrome was admitted. Multiple pulmonary infiltrates were revealed on a chest computed tomography scan, which progressively worsened even after chemotherapy and corticosteroid therapy. No evidence of infection was observed in bronchoalveolar lavage fluid. A histological examination of a transbronchial lung biopsy specimen showed lymphocyte invasion with fibrosis, indicating that the pulmonary infiltrates were OP associated with MDS. Before transplantation, she suffered from respiratory failure and required oxygen supplementation. She developed idiopathic pneumonitis syndrome on day 61 that responded well to corticosteroid therapy, and the OP pulmonary infiltrates improved gradually after HSCT, She was discharged on day 104 and is well without recurrence of OP or MDS 2 years after HSCT.

## Introduction

Pulmonary complications in patients with hematologic malignancies are due mainly to infections from their neutropenic status. However, pulmonary complications are occasionally associated with underlying malignant diseases, including leukemic cell infiltration, Sweet’s syndrome, organizing pneumonia (OP), or pulmonary alveolar proteinosis. OP associated with hematologic disorders has been reported in patients with acute leukemia, Non-Hodgkin’s lymphoma, and myelodysplastic syndrome (MDS) (Cordier 2000), although the frequency is quite rare. Daniels et al. reported that there were only 11 patients among 17,808 patients (0.06%) (Daniels et al. 2007). They also reported that OP in patients with these hematologic diseases responds favorably to corticosteroid therapy, however, treatment of underlying disease is needed for patients with steroid-refractory OP.

Here, we report a case of life-threatening steroid-resistant OP associated with MDS that was also refractory to chemotherapy. We successfully performed allogeneic hematopoietic cell transplantation (HSCT).

## Case report

**Figure 1:**
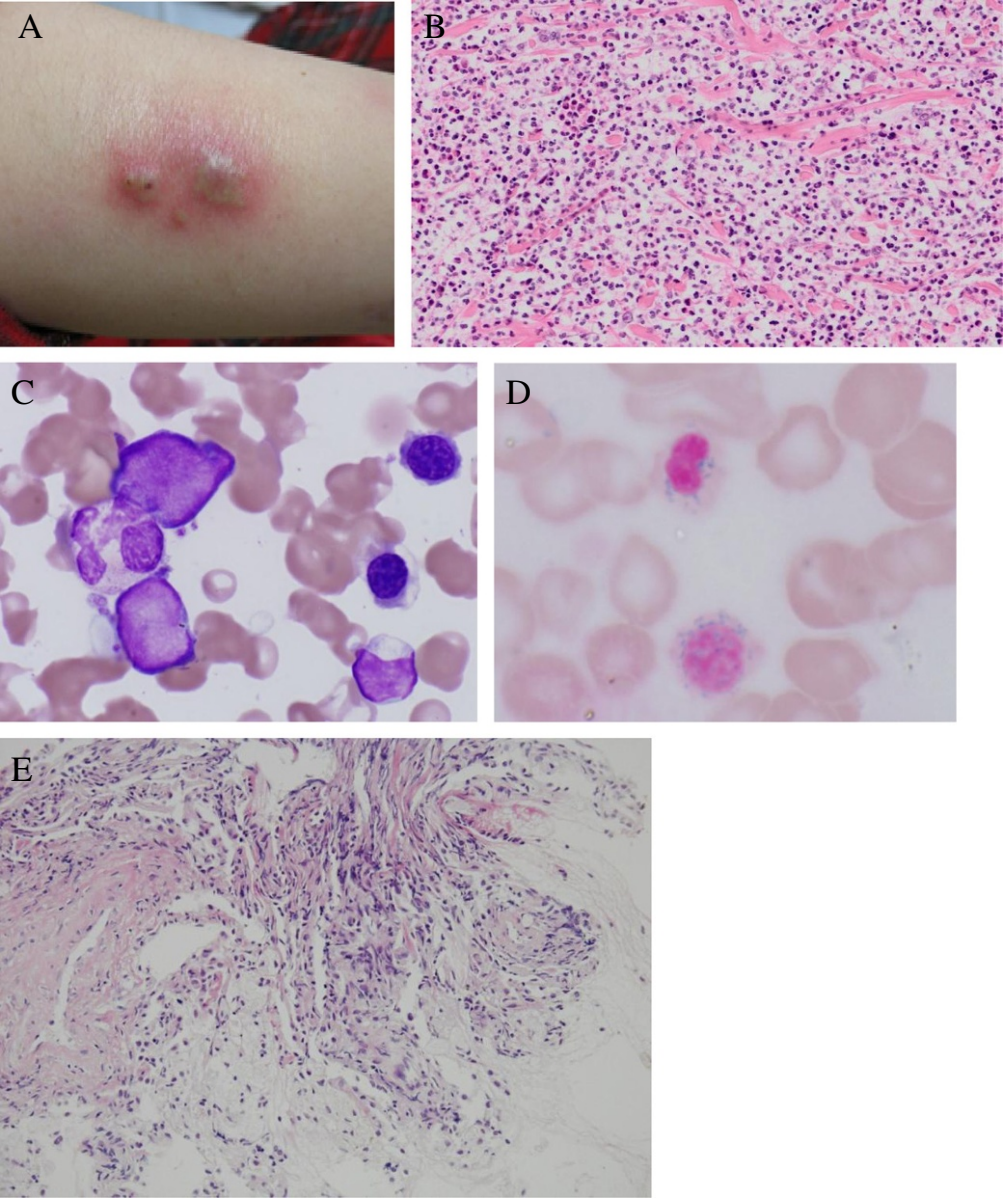
**Clinical and histopathological presentation. A**. Erythemic nodules with pustules on the right upper arm. **B**. Hematoxylin-eosin stain of the erythemic nodules: Dense dermal neutrophilic infiltrates with edema. Necrotizing vasculitis is not observed. **C**. May–Giemsa stain of the bone marrow. (×1,000). **D**. Iron stain of a bone marrow smear. Ringed sideroblasts constituted 85% of erythroid precursors. (×1,000). **E**. Hematoxylin-eosin stain of transbronchial lung biopsy material. Lymphocyte invasion accompanied with fibrosis was observed. (×100).

**Figure 2:**
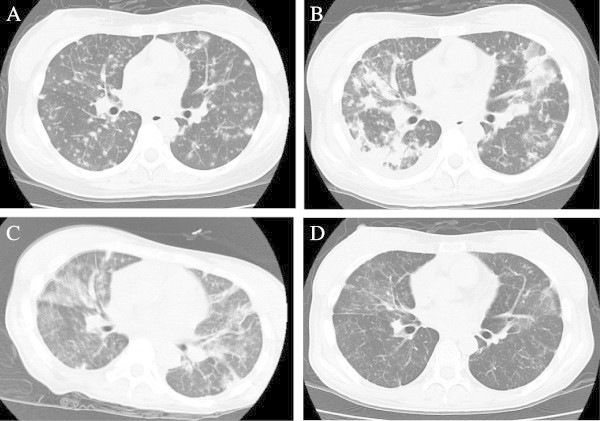
**Chest computed tomography scan. A**. Scan on admission. **B**. Scan during chemotherapy (on day-28 before allogeneic transplantation). **C**. Scan on day +61 after allogeneic transplantation. **D**. Scan on day +195 after allogeneic transplantation.

A 33-year-old female was admitted to our hospital with high fever, general fatigue, and rapidly worsening systemic erythemic nodules with pustules (Figure 1A). She had a 10-year history of MDS, refractory anemia with ringed sideroblasts (RARS), and concurrent Sweet’s syndrome; however, no systemic chemotherapy for MDS had been administered in other hospital. She had been treated only with intermittent topical and oral corticosteroid therapy for Sweet’s syndrome. On admission, a complete blood count examination revealed mild leukocytosis and anemia (white blood cells, 8.79 × 10^9^ cells/L; hemoglobin, 8.1 g/dl; hematocrit, 25.8%; red blood cells, 2.77 × 10^12^ cells/L; platelets, 15.3 × 10^9^ platelets/L). Peripheral blood smears showed that 32.5% of the cells were erythroblasts with a marked left-shift in neutrophils (myeloblasts, 3.5%; myelocytes, 4%; metamyelocytes, 1.5%; bands, 22%; segments, 29%) with 1.0% eosinophils, 2.5% basophils, and 36.5% lymphocytes. A bone marrow examination revealed hypercellularity with 62.3% erythropoietic cells, 23.6% granulopoietic cells, and 2.3% blasts (Figure 1C). Ringed sideroblasts constituted 85% of the erythroid precursors (Figure 1D). A cytogenetic analysis revealed 46, XX, +1, der (1;7)(q10; p10) and del (20)(q11.2q13.3). A skin biopsy reconfirmed dense dermal neutrophilic infiltrates with edema without necrotizing vasculitis in hematoxylin-eosin-stained sections (Figure 1B). A diagnosis of refractory anemia with excess blasts-1 that had progressed from RARS with concurrent Sweet’s syndrome was made. A chest computed tomography (CT) scan on admission revealed multiple bilateral nodules suspicious of a fungal infection (Figure 2A). Although tests based on several techniques for detecting a fungal infection, including galactomannan, (1,3)-β-D-glucan, *Aspergillus* antigen, and cultures of various tissues were negative, we started voriconazole and broad-spectrum antibiotics.

She was treated with a single course of combination chemotherapy consisting of aclarubicin, Ara-C, granulocyte colony-stimulating factor, and two courses of azacitidine. The effects of the chemotherapy were considerably limited and her clinical symptoms, including high fever and skin nodules, did not improve. Additionally, her pulmonary infiltrates progressively worsened during chemotherapy (Figure 2B). Methylprednisone (mPSL) pulse therapy was administered; however, the pulmonary infiltrate condition did not improve. Fiber-optic bronchoscopy and a bronchoalveolar lavage (BAL) were performed to characterize the pulmonary infiltrates. The BAL fluid included 19 × 10^4^ cells consisting of lymphocytes (77%), macrophages (23%), and few or no neutrophils (0%). A lymphocyte subset demonstrated that 83.2% of the lymphocytes were CD3^+^ T cells and the ratio of CD4^+^: CD8^+^ T cells was 0.65. No bacteria or fungi were seen on Gram-stained preparations or in BAL fluid cultures. A histological examination of material obtained by transbronchial lung biopsy (TBLB) showed lymphocyte invasion accompanied by some fibrosis (Figure 1E).

Therefore, it was likely that the pulmonary infiltrates were OP associated with MDS; thus, she received an allogeneic peripheral blood stem cell transplant (PBSCT). The donor was human leukocyte antigen (HLA)-A serological and HLA-A and B allele mismatched mother because she had no alternative donor source. The preparative regimen consisted of fludarabine (30 mg/m^2^) for 5 days and busulfan (3.2 mg/m^2^) for 2 days. Graft-versus-host disease prophylaxis was initiated with tacrolimus and short-term methotrexate. The tacrolimus was changed to cyclosporine A on day 57 due to renal dysfunction. Prefrozen peripheral blood stem cells contained 4.4 × 10^6^ cells/kg CD34^+^ cells were administered. The absolute neutrophil count recovered to 0.5 × 10^9^ neutrophils/L on day 15 and the platelet count reached 50 × 10^9^ platelets/L on day 17. Using the short tandem repeat-polymerase chain reaction method, we confirmed that the chimerism of peripheral blood CD3^+^ T lymphocytes was complete (100%) donor type on day 20. A bone marrow examination was performed on day 34, and cytogenetic remission was confirmed by fluorescence *in situ* hybridization. Although pulmonary infiltrates on chest X-ray improved gradually, she complained of respiratory distress on day 61 (Figure 2C). A chest CT scan indicated ground-glass opacity, and an arterial blood gas (ABG) analysis indicated carbon dioxide narcosis; thus, she required short-term (10 days) mechanical ventilation. Fiberoptic bronchoscopy was performed, and her BAL fluid included macrophages and lymphocytes. No bacteria or fungi were seen on Gram-stained preparations or in BAL fluid cultures. We concluded that the etiology of the pulmonary distress was idiopathic pneumonitis syndrome. The patient received three doses of 250-mg mPSL mini-pulse therapy and the ABG and chest X-rays of the pulmonary infiltrates improved remarkably. On day 9 of mPSL administration, she did not require O_2_ supplementation, and a chest CT scan on day 12 of mPSL administration revealed a remarkable improvement in ground-glass opacity. Finally, she was discharged on day 104 after HSCT. A bone marrow examination on day 153 after HSCT demonstrated complete remission of the MDS, and no Sweet’s syndrome was observed. A follow-up chest CT scan on day 195 (Figure 2D) revealed that no pulmonary infiltrates or ground-glass opacity had recurred. She is currently well without recurrence of OP or MDS 2 years after HSCT.

## Discussion

Pulmonary complications in patients with hematological malignancies are most often caused by bacterial or fungal infections; therefore, we treat patients with broad spectrum antibiotics and/or antifungal agents as first-line treatment (Wingard et al. 2012). In addition, patients can occasionally suffer from heart failure or pulmonary hemorrhage due to cytopenia or the effects of anticancer drugs. Nevertheless, previous reports have demonstrated that pulmonary infiltration can be associated with an underlying disease, including leukemic cell infiltration, Sweet’s syndrome, pulmonary alveolar proteinosis, or OP (Tenholder et al. 1990; Stemmelin et al. 1991; Drent et al. 1997; Daniels et al. 2007). A TBLB and BAL are the most useful methods for diagnosing such conditions, including leukemic cell infiltration or pulmonary alveolar proteinosis. However, the differential diagnosis of Sweet’s syndrome and OP is difficult.

Basically, the pathological difference between pulmonary Sweet’s syndrome and OP is clarified by the invaded cell types. Sweet’s syndrome of the lung usually shows interstitial edema with alveolar exudate of the neutrophils (Takimoto et al. 1991). On the other hand, increased lymphocytes with other cells including neutrophils, eosinophils, plasma cells,or mast cells are seen in OP (Cordier 2000). Diagnosis is further confirmed if buds of granulation tissue were detected (Reid et al. 1996). Unfortunately, buds of granulation tissue were not identified in the TBLB sample. However, BALF findings strongly indicated that our case was OP.

The first-line treatment for OP associated with MDS is corticosteroid therapy (Cordier 2000; Daniels et al. 2007). Many reports have suggested the efficacy of corticosteroids; however, the pulmonary infiltration in our patient was refractory to corticosteroids, and severe respiratory distress was observed. Additionally, the status of MDS had progressed. Based on these findings, we performed allogeneic PBSCT to treat both the MDS and MDS-related OP. It is possible that her OP responded to calcineurin inhibitors because immunosuppressive drugs are one of the alternative treatment against OP (Cordier 2000). Lee et al. reported that cyclosporine with macrolide were used for the treatment of steroid-refractory OP (Lee et al. 2011). Further study about treatment strategies for steroid-refractory OP is warranted.

Problems with allogeneic HSCT for patients with pulmonary complications include transplant-related mortality. Pulmonary comorbidities are the most prevalent in the hematopoietic cell transplantation (HCT)-specific comorbidity index (HCT-CI), (Sorror et al. 2005). The patient’s respiratory status, complaints of dyspnea at rest, and requirement for O_2_ at the time of transplantation indicated severe pulmonary comorbidity that merited the highest score. The presence of pulmonary disease in MDS patients is an independent prognostic factor in a univariate analysis and HCT-CI is prognostic impact-independent of the International Prognostic Scoring System (Zipperer et al. 2009). In contrast, Tabata et al. reported successful allogeneic HSCT for a patient with MDS and severe pulmonary alveolar proteinosis. Although it was difficult to decide to perform allogeneic HSCT for our patient, we successfully performed the procedure in a patient with MDS and severe pulmonary complications. In conclusion, allogeneic HSCT should be considered a treatment option if pulmonary complications are definitely associated with an underlying hematological malignancy.

## Consent

Written informed consent was obtained from the patient for the publication of this report and any accompanying images.
